# Astrocyte-Derived Extracellular Vesicles (ADEVs): Deciphering their Influences in Aging

**DOI:** 10.14336/AD.2021.0608

**Published:** 2021-09-01

**Authors:** Megan E Rouillard, Pearl A Sutter, Olivia R Durham, Cory M Willis, Stephen J Crocker

**Affiliations:** ^1^Department of Neuroscience, University of Connecticut School of Medicine, Farmington, CT 06030, USA.; ^2^Department of Clinical Neurosciences and NIHR Biomedical Research Centre, University of Cambridge, Cambridge, UK.

**Keywords:** aging, exosome, p16, myelin, neurodegeneration, extracellular matrix, regeneration

## Abstract

Astrocytes are an abundant and dynamic glial cell exclusive to the central nervous system (CNS). In the context of injury, inflammation, and/or diseases of the nervous system, astrocyte responses, termed reactive astrogliosis, are a recognized pathological feature across a range of conditions and diseases. However, the impact of reactive astrogliosis is not uniform and varies by context and duration (time). In recent years, extracellular communication between glial cells via extracellular vesicles (EVs) has garnered interest as a process connected with reactive astrogliosis. In this review, we relate recent findings on astrocyte-derived extracellular vesicles (ADEVs) with a focus on factors that can influence the effects of ADEVs and identified age related changes in the function of ADEVs. Additionally, we will discuss the current limitations of existing experimental approaches and identify questions that highlight areas for growth in this field, which will continue to enhance our understanding of ADEVs in age-associated processes.

Astrocytes are a prominent and plentiful glial population found in the central nervous system (CNS). Astrocytes perform homeostatic functions critical for the proper functioning of the CNS, including modulating neuronal metabolism and activity [1, 2] and maintenance of the glia limitans and the blood brain barrier (BBB) [3-5], which occurs through astrocytic end feet that extensively cover the vasculature of the brain to regulate the exchange of nutrients, metabolites, and other materials between blood and brain parenchyma [6]. Astrocytes also directly communicate with other glial subtypes, such as microglia and oligodendrocytes [7-9], in addition to neurons [10-12]. These functions can be disrupted by injury, infection, or disease which ultimately lead to altered astrocyte behavior and widespread disruptions of CNS homeostasis [13, 14]. The loss of homeostatic control following reactive astrogliosis can have opposing effects to either exacerbate disease or mitigate it; the nature of the response is highly dependent on the nature of the insult, and the interplay between its many factors [4, 15]. The multitude roles of astrocytes in modulating the normal functioning of the CNS do not always require physical cell-to-cell contacts. An increasing number of studies now point to astrocyte-derived extracellular vesicles (ADEVs) as an important facet of this influence of astrocytes on surrounding tissue. In fact, astrocytes secrete a wide-array of factors under both physiologic and disease-reactive states. In this review we will present the current state of knowledge on the contribution of EVs to the astrocyte secretome. This is a topic of emerging interest and increasingly appreciated as a means of communication by which astrocytes can affect other cells. In particular, we will discuss how ADEVs are an important functional component of astrocyte associated responses to age. This rapidly evolving concept of EV-mediated communication among astrocytes represents a new mode of action by which astrocytes may exert influence over physiological processes in health and disease.

## Extracellular Vesicles

Extracellular vesicles (EVs) were first described as “platelet dust” over 50 years ago [16]. Over the following decades, what was initially referred to as “dust” has now emerged as bona fide cellular-derived vesicles that are actively produced and released from virtually all cell types, which can be identified in all biological fluids examined, from cell culture media to blood and cerebrospinal fluid [17]. Morphologically, EVs consist of a lipid bilayer that encapsulates a small volume of cytosol. Cargo is transported in the aqueous center or embedded in the lipid bilayer [18, 19]. EV cargo can consist of diverse biological material such as nucleic acids, proteins, and lipids [20] in addition to mitochondria [21]. The diversity of molecules that make up EV cargo is best represented by a high-throughput screen that identified approximately 3,500 distinct proteins and 2,000 lipid species in EVs released from both cancerous and non-cancerous cells [22].

There are three main types of EVs classically described in literature, as defined by their mechanism of release and size: exosomes, microvesicles and apoptotic bodies [23]. Apoptotic bodies are generally larger than 100 nm, released directly from the plasma membrane of cells undergoing apoptosis and will not be discussed further here, as the majority of ADEVs are released from cells not undergoing apoptosis. Exosomes have a diameter between 30 - 150 nm and are released into the extracellular environment after the fusion of multivesicular bodies (MVB) with the plasma membrane [23]. Briefly, exosomes are created when early endosomes mature to late endosomes, also called MVBs. Part of this process involves the invagination and pinching off of the endosome membrane which generates intraluminal vesicles (ILVs) [24]. Their contents are diverse and vary based on cell type as well as physiological state. The mechanism by which cargo is targeted to MVBs and sorted into ILVs is not well understood, but it is likely not a random process as the cargo within EVs is often present in different concentrations than in the parent cell [25]. Several studies point to the possibility that post-translational modifications of proteins may also play a role in regulating cargo selection (reviewed in [26]). While the term ‘exosome’ is frequently used to describe the pellet contained after high-speed ultracentrifugation (i.e., greater than 100,000 x *g*), this pellet may also include microvesicles as well as non-membrane bound proteins [23, 27].

Microvesicles and exosomes are similar in form and function, yet they differ in subtle ways [28]. While both transport nucleic acids, proteins, and lipids through the extracellular space, microvesicles are generally larger than exosomes, with diameters ranging between 100 nm - 1 μm [20]. Microvesicles are generated by the outward blebbing of a cell's plasma membrane before it is pinched off and released into the extracellular environment [29], [30]. Like exosomes, microvesicles selectively recruit certain cargo while excluding others [25, 31].

Currently, the International Society for Extracellular Vesicles (ISEV) recommends using the generic term EV over the terms linked to biogenesis, as determining the mechanism of origin is difficult unless live imaging of EV release will be done [32]. EV refers to particles released from the cell with a lipid bilayer, and incapable of replication due to lack of a functional nucleus. Instead, using size, biochemical markers, condition or cell of origin to delineate different types of EVs is recommended [33]. Therefore, in this review, we will broadly define particles as EVs in line with current recommendations, even if the primary publications characterized the vesicles as exosomes or microvesicles.

The release of EVs from cells was once thought to only be a means for the disposal of cellular waste or components. It is now known that EVs fill many more roles than waste disposal, and are currently being investigated for their potential as a noninvasive diagnostic tool to assay patient urine [34] and/or blood samples [35] [36] in cancer [37] and autoimmune diseases [38, 39] along with many other diseases [40, 41]. The main focus of this review, however, centers on EVs secreted from astrocytes as a biologically active signaling mechanism whereby astrocytes, either through directed or indirect release of EVs, exert an effect via fusion with a target cell. Some work has shown that EVs from certain cell types preferentially interact with other cell types, such as B cell-derived EVs selectively binding to follicular dendritic cells [42] and EVs released from T84 human intestinal epithelial cells preferentially binding to dendritic cells compared to T or B lymphocytes [43]. Dendritic cells can also release EVs with MHC class II and other stimulatory molecules that can lead to T cell activation [44]. Not only have EVs been found to exert their influence on other cells via surface proteins, they also transfer genetic material such as messenger RNA (mRNA) [45], wherein cells that receive and uptake the mRNA-laden EVs can then actively translate this mRNA into proteins [46]. However, the mechanism(s) that underlie the cellular targeting of EVs are presently poorly understood [47].

## ADEVs Facilitate Intercellular Communication

Astrocytes are uniquely positioned within the CNS to both communicate with resident CNS cells and peripheral cells and tissues. It has been shown that EVs can cross the BBB [48, 49], and ADEVs have been found to be present in the CSF [50, 51], and have been found to play many important roles in the CNS. Rat astrocytes have been found to release EVs *in vitro* that contain neuroprotective and neurotrophic factors [50, 52] that actively promote neurite outgrowth [50], neuronal survival [53], and regulate production of machinery essential for synaptic function [53]. This could represent an important functional response of astrocytes in CNS injury or disease whereby their EV cargo can be altered to contain neuroprotective factors that can promote the survival of neurons and stimulate neurite outgrowth [53, 54]. For instance, ADEVs have been shown to contain a variety of proteins with important neurotrophic and neuroprotective effects, including fibroblast growth factor-2 (FGF-2), vascular endothelial growth factor (VEGF), apolipoprotein-D (Apo-D), and heat shock protein 70 (HSP70) [52, 55, 56]. FGF-2 and VEGF are angiogenic factors known to mediate proliferation, axonal regeneration, neurogenesis, synaptogenesis, and synaptic plasticity [57-60]. Apo-D and HSP70 both mediate neuroprotective effects in the CNS following injury or in disease by increasing the survival and functional integrity of neurons undergoing oxidative stress [55], contributing to myelination and remyelination processes [61], and activating microglia to promote beneficial immune-regulatory responses [62]. Previous studies have shown that the antioxidant and anti-inflammatory roles of Apo-D might hold therapeutic promise for many neurological diseases and disorders including Alzheimer’s disease, stroke, and schizophrenia, as well as neuroprotection against aging [63-66]. Similarly, astrocytes exposed to the inflammatory cytokines interleukin (IL)-1β and tumor necrosis factor (TNF)-α *in vitro* were found to increase the release of ADEVs containing high levels of specific micro RNA (miRNA) species that promoted neuronal survival through decreased neuronal activity [54]. Further, astrocytes exposed to the anti-inflammatory cytokine IL-10 or adenosine triphosphate (ATP) *in vitro* resulted in the release of ADEVs containing proteins known to promote neurite outgrowth, dendritic branching, synaptic transmission, and neuronal survival [53]. ADEVs have also been found to exert protective effects in other stress conditions, such as artificially induced heat shock [56], excitotoxicity [67], and oxidative stress [55]. Hence, ADEV release is not a static process, rather it is a highly adaptive and dynamic process which is central to the recognized role for astrocytes as important mediators of brain homeostasis [68, 69]. ADEVs are only one way in which astrocytes and neurons interact, but the full extent of astrocyte-neuron interaction is beyond the scope of this review and has been extensively reviewed elsewhere (see [70] and [71]).

While research on the interaction between ADEVs and other glia is still in its infancy, there have been some studies that suggest ADEVs also have an impact on these cells. ADEVs from activated astrocytes were found to promote microglial transformation towards an anti-inflammatory phenotype in murine models of traumatic brain injury [72]. In mouse models of ALS, SOD1 mutant astrocytes release EVs that increase microglial expression of inducible nitric oxide synthase (iNOS) and TNF-alpha. These changes are associated with microglial activation, but the ADEVs were also found to induce apoptosis of microglia [73]. It is well recognized that microglia are important activators of astrocyte responses in a range of human neurological diseases, these ADEV focused studies also indicate a reciprocal communication from astrocyte to microglia, which may have similar influence and impact on neuropathology and function.

It is equally important to note that astrocytes are capable of communicating with cells over large distances in both the CNS and the periphery using EVs [74]. From this, ADEVs have also been implicated in mediating neuroinflammatory responses. A recent study from Dicken and colleagues showed that mice given an intracerebral injection of IL-1β, a model of inflammatory brain injury, led to reactive astrogliosis and the release of ADEVs that promoted and directed leukocyte movement to the site of injury through modulation of the peripheral cytokine response [75]. These data point to the potentially exciting prospect that ADEVs actively influence not only CNS inflammation, but also peripheral immune response during injury and disease. This represents a paradigmatic shift in our thinking for many neurological diseases where the CNS, rather than passively reacting to injury or disease, instead takes an active role through the systemic release of ADEVs to modulate and direct the response of peripheral immune cells to the CNS. The interaction between the CNS and the immune system has been studied in many diseases, including multiple sclerosis, Alzheimer’s disease, Parkinson’s disease, and amyotrophic lateral sclerosis (ALS) [76], but the role of ADEVs in this capacity has yet to be thoroughly examined. Collectively, these studies have demonstrated that ADEVs can affect the outcomes of damaging events by fortifying neurons through the release of neuroprotective factors and simultaneously modulating peripheral immune responses to that injury. These exciting possibilities lead to questions about the nature of cues that specifically stimulate the compartmentalization of these factors as cargo into ADEVs for release. Future research investigating the conditions and processes that manage ADEV content are critical to furthering the understanding of ADEV function. Whether ADEVs in complex biological fluids, such as blood or CSF, could be assayed to better monitor the specific CNS response to pathology and offer a biomarker potential with therapeutic value are areas of active investigation.

## ADEVs are Implicated in Aging and Senescence

One of the primary risk factors for the development of neurological diseases is age [77]. With chronological aging widespread changes occur from the tissue level down to the cellular and even molecular level. While many of these changes are currently thought to be mild and benign, a growing consensus among researchers is that many of the tissue, cellular, and molecular alterations observed in aging leads to the loss of cellular resistance that renders cells more susceptible to damaging stimuli, such as oxidative stress [78], inflammation [79-81], metabolic dysfunction [82, 83], and the accumulation of DNA damage [84, 85]. Further, sequencing of murine glia identified significant changes to the transcriptome and epigenome of these cells in the course of natural aging [86, 87]. It has been shown that astrocytes change their transcriptional patterns with age in a region dependent manner [87]. Comparing gene expression in young and aged murine astrocytes showed aged astrocytes developed a pro-inflammatory phenotype through up-regulation of genes associated with reactive astrogliosis. Determination of the cell types critically affected and intimately involved in this process are under intense study.

Under diseased conditions, astrocytes become alternatively activated, which impairs the normative functions of astrocytes and leads to a reactive, pro-inflammatory state termed reactive astrogliosis [4, 88]. Reactive astrogliosis is a prominent feature found in every chronic neurodegenerative disease and is increasingly recognized as a determinant of tissue dysfunction in aging [89, 90]. Here, chronological aging is associated with the development of a CNS pro-inflammatory status that is attributed to the development of cellular senescence predominantly among glial cells [91].

Cellular senescence is classically defined as the irreversible exit of a cell from the cell cycle or put simply, permanent arrest of cellular growth. This prohibits the proliferation of cells exposed to oncogenic stressors, including DNA-damage [84, 85], shortening telomeres [92], or irradiation [93]. This is thought to be the result of tumor-suppressor activation [1, 94]. However, the concept of senescence has expanded and grown since these initial definitions and has emerged as a complex phenomenon occurring in a range of biological processes, from embryonic development to wound healing, tissue repair, and organismal aging [95, 96]. An important biological outcome of senescent cell formation is the development of the senescence-associated secretory phenotype (SASP) [97, 98]. The SASP is both cell- and context-dependent and demonstrates the ability of senescent cells to express and secrete a heterogeneous array of extracellular modulators, including cytokines, chemokines, proteases, growth factors, and bioactive lipids [99] [97]. Here, the SASP is thought to reinforce senescence-induced cell cycle arrest, stimulate immune-mediated clearance, and limit scar formation to promote wound healing and tissue regeneration [100, 101]. Senescence has also been shown to increase EV secretion [102] regardless of how senescence is induced[102] [103]. Senescence-associated EVs have been implicated in the DNA damage response [103], cancer cell proliferation [104], calcification of blood vessels [105], decreased myelination, [106] and immune cell activation [107-109]. Senescence-associated EVs can contain varied fragments of both single and double stranded DNA [110, 111] and is a major route of DNA secretion in senescent cells [103]. Here, the secretion of harmful DNA within EVs increases during senescence [1]. There is now strong evidence that not only do astrocytes become senescent in normal aging and disease, they also develop a robust SASP [112, 113].

**Figure 1.**
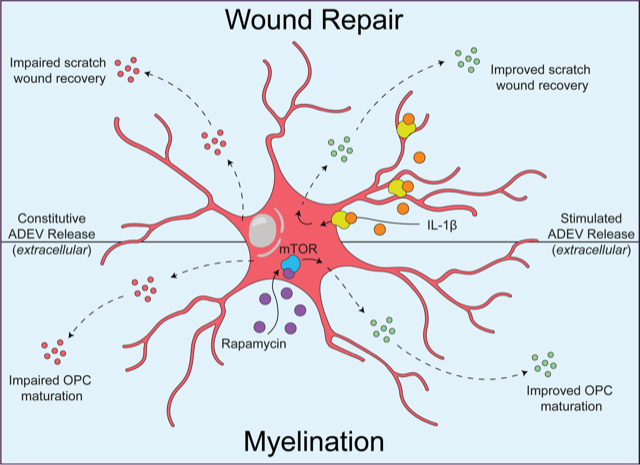
Schematic representation of an astrocyte summarizing the diverse responses and functional impact of ADEVs under constitutive and stimulated release in aging.

Astrocytes are uniquely positioned in the CNS to interact with and influence a wide-range of cellular networks, such as neuron-neuron connections, microglial activity, and maintenance of oligodendrocytes and myelin through secreted extracellular factors [114] (Fig 1). This unique integration among the critical networks of the CNS means dysfunctional astrocytes can negatively impact CNS function on a large and devastating scale [4, 88] [115, 116]. Specifically, in aging and in senescent astrocytes, it is becoming increasingly clear that ADEVs are an important component of the SASP. Recent work has shown that aged (and senescent) astrocytes release EVs laden with factors that vary widely based on context and cell type. These findings offer the potential for astrocytes to contribute to damage or repair in aging and disease [117, 118].

In a recent study, primary murine astrocytes were cultured long-term (>12 weeks *in vitro*) to induce the development of a senescent phenotype as defined by the increased expression of canonical senescent gene markers *p16^INK4A^*, *p21*, and *p53* in parallel with increased senescence-associated beta galactosidase (SA-β gal) staining when compared to primary murine astrocytes cultured short-term (<4 weeks *in vitro*) [117]. EVs captured and isolated from both long- and short-term cultures showed a marked difference in their respective proteome. When ADEVs from long-term cultures were applied to rat oligodendrocyte progenitors (rOPCs) there was a near complete inhibition of their differentiation into mature, myelin-producing oligodendrocytes that was not observed in rOPCs treated with ADEVs from short-term cultures. Despite the widespread consideration of astrocytes as drivers of neurodegenerative disease progression, this is perhaps the first study to identify the functional impact of aged (or senescent) astrocytes on myelin integrity and myelination itself [118]. However, these dysfunctional astrocytes are not permanently locked into this alternative state. Within this same study, the authors found that treatment with the mechanistic target of rapamycin (mTOR) inhibitor rapamycin diminished the impact of the senescence state on the cells as well as altering the ADEV proteome. When ADEVs from rapamycin-treated long-term murine astrocyte cultures were applied to rOPCs, an increased percentage of mature, myelin producing oligodendrocytes were observed [118]. This indicates that, although aged astrocytes are seemingly incapable of supporting the *de novo* generation of myelin through the released of ADEVs, they are permissive to therapeutic intervention to improve their trophic support of other glial cells.

Paradoxically, senescent cells have been found to improve wound repair by limiting fibrosis [100]. As major producers of the extracellular matrix that leads to fibrotic scar formation in CNS injury and disease [119], how astrocytes contribute to this process has only recently been explored. Here, both short- (i.e., young) and long-term (i.e., aged) murine astrocyte cultures showed no deficits in their ability to recover from a scratch-wound [117]. However, aged astrocytes treated with only the ADEV fraction isolated from aged astrocytes significantly reduced their recovery following a scratch wound when compared to aged astrocytes that did not receive ADEVs. Within the same paradigm, the recovery of young astrocytes following a scratch wound was not impacted following treatment with ADEV fraction isolated from young astrocytes. Follow-up heterochronic experiments where ADEVS isolated from young astrocytes were administered to aged astrocytes with a scratch wound (and vice versa) further supported the inhibitory impact of aged astrocytes on wound repair [117]. This finding further reinforces the view of an altered ADEV proteome with the context of aging that can impact not only the differentiation potential of oligodendroglial lineage cells but can act in both an autocrine and paracrine manner to directly influence astrocyte responses to injury. Interestingly, when the authors pre-treated aged astrocytes with the pro-inflammatory cytokine IL-1β (to recapitulate the inflammatory microenvironment of a wound), ADEVs from the cytokine-treated aged astrocytes significantly improved wound recovery. Whereas, IL-1β pre-treatment of young astrocytes produced ADEVs that inhibited wound repair in all conditions. In line with the previous finding with rOPCs, rapamycin treatment of aged astrocytes led to the production of ADEVs with pro-wound healing properties [117].

These findings indicate that despite increasing age having a negative impact on the function of astrocytes, under the right context there is a beneficial, yet limited response to tissue injury. Additionally, aged astrocytes are amenable to therapeutics that suppress the senescent phenotype to restore aspects of astrocyte trophic support for other glial cells. This will be important moving forward as additional anti-aging and senescence suppressing drugs are investigated in both *in vitro* and *in vivo* conditions. Further, these findings strengthen the view that ADEVs are critical components of the SASP within senescent astrocytes and require further investigation into their beneficial and/or detrimental roles in both healthy and unhealthy aging.

## Considerations for advancing the study of ADEVs

From this discussion, we have connected the burgeoning field of astrocyte communication to that of ADEV release. These studies refine our understanding of their roles within an organism that could be considered orders of magnitude more complex than current approaches can rigorously study. Nevertheless, the anticipated impact of understanding the functions of ADEVs in disease is supported by the widening scope of work in this field. This prompts us to consider several salient questions which have naturally emerged about the potential impact and utility of ADEVs.

### Can ADEVs be prognostic or diagnostic for identifying disease?

ADEVs have been implicated in an increasing number neurological and degenerative diseases. Their functions in these conditions are not always clear. For instance, astrocytes in Alzheimer’s disease uptake amyloid-β and can release ADEVs, along with other pathogenic factors leading to increased neurodegeneration and neuroinflammation. ADEVs contain high levels of complement proteins, which contribute to ongoing neuroinflammation [120]. This is also correlated with diminished levels of complement regulatory proteins in preclinical AD patients [120], which may have an effect on the phagocytic activities of microglia [121]. Similarly, a recent paper revealed that ADEVs can play a harmful role in an experimental animal model of stroke. Following occlusion of the middle cerebral artery in rats, ADEVS were overly abundant with SEMA3A, an extracellular matrix (ECM) protein that promotes astrocytic activation and leads to reduced axon growth and inhibition of repair following ischemia [122]. In traumatic brain injury (TBI), increased levels of neurotoxic complement proteins are present in ADEVs found in blood plasma. Some proteins were not normalized even years after injury, potentially explaining the long lasting post-concussive symptoms many patients experience. These ADEVs have the potential to be used as a diagnostic tool to determine severity of disease and monitor recovery. This also suggests that TBI patients might benefit from complement inhibitors, but more study is needed [123]. Similarly, we have previously determined that ADEVs can be identified in murine blood plasma and the proportions of ADEVs are increased in the induced autoimmune encephalomyelitis (EAE) model of neuroinflammation [124]. With the changes in ADEV functions with age, and elevated basal levels of inflammation recognized in the aged CNS, it may be expected that plasma ADEVs may also change with age, including yet to be defined prognostic indicator(s) of neurological health or disease.

### What process(es) determine the cargo of ADEVs?

A pivotal question in the field of EV biology is how does a cell ‘decide’ what to package into EVs for extracellular communication or distribution? It is now known that the contents of ADEVs vary, and when searching available databases of proteomic libraries of ADEVs one will find a wide range of potential EV cargo that differ with disease state, cellular experience, or age. Factors that impart influence on ‘the decision process’ can include intracellular and extracellular cues. Recently, we have reported that the fundamental experience of astrocytes simply determined by the ECM upon which they are cultured has a dramatic influence on the ADEVs those astrocytes generate [125]. This was demonstrated by applying these ADEVs from astrocytes cultured on various ECM substrates onto naive astrocytes which uniquely modified the responses of the naive astrocytes both in terms of mechanical or inflammatory challenges [125]. Similarly, we have shown that ADEVs derived from aged astrocytes in culture lose their trophic function to support the maturation of oligodendrocytes, which is robust with ADEVs from young astrocytes. Similarly, the genetic phenotype of astrocytes also impacts ADEV functions. Previous studies have indicated that ADEVs released in ALS mouse models can contain mutant superoxide dismutase (SOD) 1, which can be transferred to spinal neurons, selectively inducing motor neuron death [126]. The involvement of ADEVs in ALS pathogenesis was further supported by a recent paper by Sproviero and colleagues who found that ADEVs carry mutated transactive response DNA binding protein (TDP-43), a nuclear protein that, when mutated, is known to aggregate and trigger neurodegeneration [127, 128] [129]. Interestingly, overexpression of mutated SOD1 in astrocytes when compared with wildtype astrocytes resulted in an overall decrease (43%) in the protein secretome, whereas the protein content of ADEVs was specifically increased (37%) [126]. These studies suggest that the oxidative stress brought about by the loss of SOD1 function fundamentally alters the secretome landscape. These, and other, demonstrations that there is an association between the ADEV cargo that is determined by conditions and experiences strongly suggest that the contents of an ADEV is not random and is likely orchestrated and purposeful. Future studies deciphering the intracellular processes that determine which intracellular factors (proteins, lipids, and/or nucleic acids) are shuttled to become the cargo for an ADEV are warranted.

### Can ADEVs be modulated in a cell specific manner?

At present there are few experimental strategies that can target the release of EVs in a cell-specific way in *in vivo* systems to test questions of the impact of specific cellular EV sources. In the context of aging, it is interesting to note that in senescent fibroblasts DNA damage activates the ceramide synthetic pathway which increases levels of EV secretion. This pathway is thought to prevent apoptosis through the removal of DNA fragments from the cytosol which protects the cell from an overactive inflammatory response [130]. It is possible that this pathway is more universal, and more work needs to be done to confirm or disprove this. Astrocytes have also been shown to release EVs with mitochondrial DNA, potentially propagating pathology in a cell-to-cell manner [131].

Experimentally, pharmacological targeting of EV trafficking and lipid metabolism has been reported to attenuate EV release from many different cell types. Therapies targeting inhibition of EV trafficking include calpeptin, manumycin A and GW4869 (reviewed in [132]). Calpeptin, an EV shedding inhibitor, has been shown to inhibit EV release in HEK293, SH-SY5Y, and PC3 cell lines as well as platelets [132], however its effect on the release of ADEVs has not been studied. Calpeptin has been shown to decrease astrocytic activation in murine models of depression [133] and neuroinflammation in a Parkinson’s disease murine model [134] indicating that although its impact on ADEV release has not been specifically studied, calpeptin holds therapeutic promise for investigating ADEV modulation.

Another means of modulating ADEV release is though the inhibition of neutral sphingomyelinase 2 (nSMase2) by GW4869, which has been reported to have efficacy in reducing pathology in a mouse model of AD [135]. However, nSMase2 is a key regulatory enzyme in the production of ceramide from sphingomyelin. Mutations in sphingomyelin phosphodiesterase 1 (SMPD1), which converts ceramide to sphingomyelin, underlie Niemann-Pick’s disease. This suggests that a delicate balance exists in the regulation of lipid metabolism, and perturbations in this balance could have unanticipated, deleterious consequences from the use of these agents beyond the experimental models. At present, the lack of specificity of these particular agents to target cellular populations is an important caveat to their use. The natural function of EV release in unaffected tissues or the homeostatic function of the enzymes inhibited may have negative effects on other tissues or cells. These drawbacks may diminish enthusiasm for these agents as potential therapeutics, but should not diminish the potential importance of these agents as experimental tools. From this perspective, future experiments employing these agents to understand the processes of EV production in general and modulating the release from astrocytes would be expected to still offer important insights into our understanding of the actions of ADEVs in certain settings. Future studies testing these questions, but employing cell-specific modifications that can alter EV release in an experimenter-controlled manner, would be considered an important advance for the field as it would introduce a new level of rigor by which to launch studies with more clinically translational applications to test ADEV functions in pathology and pathogenesis of diseases.

### Can ADEVs be adapted for therapeutic use?

The potential for exploiting or harnessing the influence of ADEVs for therapeutic use is an attractive prospect. With their small size and ability to be carried through biological fluids, they may serve as sentinels for monitoring CNS function as well as disease diagnosis, progression, or treatment efficacy. As we and others have shown, ADEVs can be influenced to modify function, yet how to coax the yet to be defined process(es) that determine cargo loading is currently unknown. However, the *ex vivo* development of ADEVs for collection and administration as a therapeutic potential is an attractive prospect, whether as a vehicle for therapeutic RNA [136] or protein [137]. Adding specific surface ligands may also be useful for targeting specific cell types or ensuring engineered EVs can cross the BBB. Some issues arise in terms of producing large enough quantities of EVs to use, but there has been success using EVs generated in plants for use in mammalian cells [138]. While no EV mediated therapies have been approved for use in humans as of yet, there are numerous treatments being developed both in *in vitro* and *in vivo* models. EVs from murine adipose and bone marrow mesenchymal stem cells (MSC) have been shown to restore synapse function, as well as improve learning and memory in mice after cerebral ischemia [139]. Pattern separation and spatial learning deficits were improved after traumatic brain injury with human bone marrow MSCs [140]. The potential for astrocyte EVs to be used to treat neurological disorders is, as of yet, largely unexplored.

### What determines the cellular targets of ADEVs?

In order for EVs to enact an intercellular signaling function, they must either interact or be taken up by a recipient cell. Possibilities for this action include EV protein docking with an extracellular receptor, direct fusion with a plasma membrane, or endocytosis followed by fusion with the endosomal membrane of a target/recipient cell [47]. As this uptake is how EVs, including ADEVs, exert influence on other cells, cellular targeting mechanisms are of great interest but are not fully understood. Several studies have shown that the EVs from certain cell types interact preferentially with other specific cell types, e.g., B cell EVs selectively binding to follicular dendritic cells [42] or intestinal epithelial cell line EVs preferentially interacting with dendritic cells over T or B lymphocytes [43]. Dendritic cells have also been shown to release EVs with MHC class II and other stimulatory molecules that activate T cells [44]. In addition to exerting influence on other cells with proteins, EVs can also transfer genetic material such as mRNA [45], and recipient cells of EVs carrying mRNA began producing the protein products coded for not long after uptake [46]. Defining the internal processes governing ADEV loading occur in astrocytes, whether they are common to other cell types or unique to glia, would be an important advance with obvious experimental and therapeutic ramifications.

### What are the current limitations of EV research?

Currently there are several technical limitations to the study of ADEVs and of EVs as a whole that are worth mentioning. One limitation of EV research is the lack of consensus on a categorization system for EV sub-classes/types in biology. This is not an issue unique to EV biology, and it is anticipated that once our fundamental understanding of the biogenesis and cargo sorting of EVs is enhanced, a system for defining EVs might evolve. Within the field of glial biology, specifically astrocytes and ADEVs, the sheer diversity of contexts under which astrocytes are involved, from development and aging to infection or disease, may mean that a strict cataloging system may be unwarranted or impractical. This issue of nomenclature was recently pointed out for proposed classification of astrocyte reactivity [141]. If definitive evidence that EVs of different size classes have clearly definable and distinct functions, then broader terms for communicating these variable functions of ADEVs may be warranted, however, the precision of language is important, and from that perspective, ADEV labeling that is linked to the nomenclature of astrocyte biology could represent a more robust scheme that would naturally evolve in concert with astrocyte biology itself.

A second limitation of the field is the availability of imaging technology used to study EVs. Many technologies currently applied in the field have advantages and disadvantages yet, overall, many of these technologies are very specialized and presently inadequate to monitor EVs with sufficient rigor. For example, flow cytometry has been used extensively in EV analysis. Indeed, while capable of capturing thousands of EVs in a single sample, and coincidently analyzing multiple surface markers, it is inherently difficult because of the physical limitations of the lasers used in standard flow cytometry. This means that the very small particle sizes of EVs and ADEVs are significantly less than the diameter of the laser beam upon which most flow cytometry applications rely. This means that additional stringencies are necessary to ensure the veracity and reproducibility of EV and ADEV analyses by flow cytometry. Without these additional steps, EVs can often go undetected in biological samples, leading to discrepancies between disparate modes of EV analysis [142]. These issues also render problematic the precision in accurate counting of EVs in samples since clustering of particles may be recorded as a single events when in high concentrations [143]. Additionally, data on EV diameters is often based on comparing EV scatter with a standard, polystyrene bead scatter. Light scattering has many factors that can affect readout other than diameter, including the shape of the object, refraction, and the absorption coefficient; this is all further compounded by lipid-based vesicles scattering far less light than the beads [144]. Some of these issues are already beginning to be resolved, with techniques such as imaging flow cytometry, combing typical flow cytometry with imaging to allow the differentiation of EVs from cells, debris, and beads, as well as increasing fluorescence sensitivity of smaller objects [145, 146]. Algorithms have also been developed to help distinguish aggregates and EVs [145]), but improvements are still needed to increase sensitivity.

Another approach to measuring EVs is dynamic light scattering: a technique that can be used to observe small particles from as small as 1 nm and up to 6 μm. This method is only reliable in monodispersed suspensions, when only one type and size of particle is present [147]. Since this is frequently not the case with EVs from biological samples, this approach currently offers little advantage to other methods. In addition, this technology is further limited by its inability to produce biochemical data or information about the cells from which the EVs were derived [148].

Nanoparticle tracking analysis (NTA) is another technology utilized for studying EVs with its own set of advantages and disadvantages. The NTA technology allows for the measurement of particles with diameters as small as 30 nm and can also be used in conjunction with fluorescence [149]. Despite the ability to employ fluorescence in NTA of EVs, limitations exist that create obstacles in the fidelity of EV analysis. The fluorescent signal must be very bright to be effectively detected by the NTA system, and there has been little success in using standard fluorescently labelled monoclonal antibodies to identify specific antigens in EVs, unless the antigen in question is exceptionally highly expressed [150]. There are, however, promising advances being made in this area involving “quantum dot” or Q-dot conjugated antibodies. This avenue still has significant obstacles to overcome, particularly it suffers from high levels of background fluorescence [151].

Perhaps the most established and reliable affirmation of EVs is through electron microscopy (EM). The utility of transmission EM (TEM) for the study and analysis of EVs cannot be overstated. For instance, TEM allows for immuno-gold labelling of EVs which affords the possibility of validating EV-specific markers [152]. However, the preparation of samples for EM can distort the native morphology of EVs. Here, cryogenic (cryo)-EM has increasingly been used to observe EVs in their natural state while avoiding the ultra-structural alterations that occur in TEM sample preparation. This is accomplished by quickly submerging the samples into cryogen (either liquid propane or ethane cooled to -196.15 Celsius) to avoid damage from sample preparation and the EM machinery itself [153]. This technique can also be used in conjunction with immuno-gold labelling [154]. In spite of these benefits and the insights afforded to the study of ADEVs using TEM, this approach is only semi-quantitative and requires both technical expertise and an EM with cryogenic capabilities.

## Concluding Remarks

We have provided a synopsis on aspects of ADEVs as a fundamental component of astrocyte biology. As outlined above, there are many new and exciting advances that implicate ADEVs in a range of pathophysiological settings. Yet, there remain many important, sometimes technical, hurdles that will be necessary to corroborate both the origins and targeting of ADEVs from astrocytes to other cells. For instance, questions of; 'why do mutated proteins always seem to find their way into EVs?' or 'can EVs from one cell target a specific cell type or subtype of another cell?', and 'how?' While technological advancements have allowed for more precise measurements and analyses of EVs, there is still a pressing need for further improvements. Improvement in technologies that enable reliable and sensitive measurement of EVs, both in their sizes and their compositions, would offer the potential to revolutionize the field by allowing for increased detail and precision. It is expected that these technical advances would translate into innovation by enabling means to answer many currently unanswerable questions about EV biology. Improvements to current technologies will allow for fundamental insights into the nature of ADEVs and how they contribute to the many facets of neurological diseases and disorders. EVs would then be poised to offer diagnostic, prognostic, and therapeutic potential within these contexts. The current foundation of our understanding of EVs would also be anticipated to generate insights into areas for which comparatively less is known about ADEVs, such as their role in pain or addiction. Thus, this rapidly evolving field will continue to garner interest as a primordial process now inextricably connected with reactive astrogliosis.?
